# Associations between Renal Hyperfiltration and Serum Alkaline Phosphatase

**DOI:** 10.1371/journal.pone.0122921

**Published:** 2015-04-08

**Authors:** Se Won Oh, Kum Hyun Han, Sang Youb Han

**Affiliations:** Division of Nephrology, Department of Internal Medicine, Ilsan-Paik Hospital, Inje University College of Medicine, Goyang, Korea; The University of Manchester, UNITED KINGDOM

## Abstract

Renal hyperfiltration, which is associated with renal injury, occurs in diabetic or obese individuals. Serum alkaline phosphatase (ALP) level is also elevated in patients with diabetes (DM) or metabolic syndrome (MS), and increased urinary excretion of ALP has been demonstrated in patients who have hyperfiltration and tubular damage. However, little was investigated about the association between hyperfiltration and serum ALP level. A retrospective observational study of the 21,308 adults in the Korea National Health and Nutrition Examination Survey IV-V databases (2008–2011) was performed. Renal hyperfiltration was defined as exceeding the age- and sex-specific 97.5^th^ percentile. We divided participants into 4 groups according to their estimated glomerular filtration rate (eGFR): >120, 90–119, 60–89, and <60 mL/min/1.73 m^2^. The participants with eGFR >120 mL/min/1.73 m^2^ showed the highest risk for MS, in the highest ALP quartiles (3.848, 95% CI, 1.876–7.892), compared to the lowest quartile. Similarly, the highest risk for DM, in the highest ALP quartiles, was observed in participants with eGFR >120 ml/min/1.73 m^2^ (2.166, 95% CI, 1.084–4.329). ALP quartiles were significantly associated with albuminuria in participants with eGFR ≥ 60 ml/min/1.73m^2^. The highest ALP quartile had a 1.631-fold risk elevation for albuminuria with adjustment of age and sex. (95% CI, 1.158-2.297, P = 0.005). After adjustment, the highest ALP quartile had a 1.624-fold risk elevation, for renal hyperfiltration (95% CI, 1.204–2.192, *P* = 0.002). In addition, hyperfiltration was significantly associated with hemoglobin, triglyceride, white blood cell count, DM, smoking, and alcohol consumption (*P*<0.05). The relationship between serum ALP and metabolic disorders is stronger in participants with an upper-normal range of eGFR. Higher ALP levels are significantly associated with renal hyperfiltration in Korean general population.

## Introduction

The glomerular filtration rate (GFR) represents the flow rate of fluid filtered through the kidney and is decided by 3 factors: glomerular blood flow, an ultrafiltration coefficient, and the hydraulic pressure difference across the kidney membrane [1]. In increased GFR, also known as hyperfiltration, these factors change, causing glomerular hypertension, subsequent glomerulosclerosis, tubular injury, and renal function decline [1,2,3]. Hyperfiltration may occur in healthy individuals, and in individuals with pathologic conditions such as diabetes, obesity, polycystic kidney disease, and focal segmental glomerulosclerosis [4,5,6,7].

Alkaline phosphatase (ALP) is a membrane homodimeric enzyme that catalyzes the hydrolysis of organic pyrophosphate [8]. ALP is present in a variety of tissues, and is especially abundant in the liver, bone, and kidney. Serum ALP level is elevated in patients with conditions that are associated with hyperfiltration, such as diabetes mellitus (DM) and metabolic syndrome (MS) [9,10]. The urinary excretion of ALP is increased in patients with compensatory hyperfiltration [11]. A small study noted that serum ALP level was higher in diabetic patients with hyperfiltration [12]. However, little was investigated about the relationship between serum ALP and hyperfiltration thus far.

We evaluated the association between serum ALP and metabolic disorders such as DM and MS, who have reduced, normal, and an upper-normal range of estimated GFR (eGFR). Next, we determined whether serum ALP is independently associated with the renal hyperfiltration.

## Materials and Methods

### Participants

The Korea National Health and Nutrition Examination Survey (KNHANES) is a nationwide representative survey on the health and nutritional status of the civilian Korean population. KNHANES is composed of a health questionnaire survey, health examination, and nutrition survey, conducted periodically by the Korea Centers for Disease Control and Prevention since 1998.

The participants for this study were chosen from the candidates, using proportional allocation-systematic sampling, with multistage stratification by age, sex, and region. The 7 metropolitan cities and 9 provinces are shown as urban and rural areas, respectively. We acquired data from the 4^th^ (IV-2,3, 2008–2009) and 5^th^ (V-1,2, 2010–2011) KNHANES. Participants in KNHANES signed an informed consent form and the survey was approved by the institutional review board of Centers for Disease Control and Prevention in Korea (IRB No. 2010-02CON-21-C). The informed consent for current study was exempted because this study was secondary analysis using the KNHANES dataset.

Among the 37,753 participants who completed the health examination, 22,376 participants aged 20 years and older, with serum creatinine and ALP data, were included in this study. Of these, 845 participants were excluded owing to liver disease (hepatitis B surface antigen seropositivity, history of liver cirrhosis, liver cancer, and hepatitis C virus infection), leaving a total of 21,308 participants for this study.

### Measurements

Blood samples, after an 8-hour fast, were collected year-round and immediately processed, refrigerated, and transported in cold storage to the central laboratory (NeoDin Medical Institute, Seoul, South Korea) for analysis within 24 hours. Routine biochemistry, including serum ALP and creatinine, was performed using the Hitachi Automatic Analyzer 7600 (Hitachi, Tokyo, Japan). Serum ALP was measured using Pureauto S ALP (Sekisui, Osaka, Japan). Serum creatinine was measured by using the Jaffe kinetic method (IV-2,3, 2008–2009; Creatinine-HR 1-Type Wako, Wako, Osaka, Japan; V-1,2, 2010–2011; CREA, Roche Diagnostics, USA). GFR was estimated by using the Modification of Diet in Renal Disease equation [13]. The 3 blood pressure (BP) readings were obtained using a mercury sphygmomanometer, and the final BP for individual participants was reported by calculating the mean of the second and third reading. Urine protein was measured by dipstick urinalysis, and the results were reported using a semiquantitative scale from negative to 4+.

### Definitions

We divided participants into 10-year age groups and renal hyperfiltration was defined as eGFR above the age- and sex-specific 97.5^th^ percentile [14,15]. Hypertension (HTN) was defined as the presence of either (i) systolic BP ≥140 mmHg or diastolic BP ≥90 mmHg or (ii) following a course of antihypertensive medication at the time of interview. DM was defined as participants who fulfilled at least one of the following four criteria: (i) fasting blood glucose ≥126 mg/dL; (ii) following a course of medication to decrease blood glucose level at the time of interview; (iii) following a course of insulin administration at the time of interview; and (iv) self-report of having received a physician’s diagnosis of diabetes. Body mass index (BMI) was calculated on the basis of weight and height (kg/m^2^). MS was defined on the basis of the National Cholesterol Education Program ATP III guidelines, only in participants with serum high-density cholesterol (HDL) cholesterol data (N = 15800) [16]. Proteinuria was defined as dipstick urinalysis above 1+. Albuminuria was defined as urine albumin creatinine ratio (UACR) ≥ 30 mg/g. Malignancy was defined as self-reported history of stomach, colon, liver, uterine cervix, breast, or other types of cancer. Myocardial infarction (MI), angina and stroke were also defined as self-reported history. Current smoking was defined as smoking on ≥1 day within the previous month. Alcohol consumption was defined as drinking ≥2 alcoholic beverages in a month within the previous year.

### Statistical analysis

All analyses were performed using SPSS software (SPSS version 20.0, Chicago, Illinois). Data are presented as the mean ± standard deviation (SD) for continuous variables and as a percentage for categorical variables. Differences were analyzed using the chi-square test for categorical variables and analysis of variance for the continuous variables. The odds ratio (OR) and 95% confidence interval (95% CI) of associated factors for DM, MS, and renal hyperfiltration were calculated by using logistic regression analysis. A P-value <0.05 was considered statistically significant.

## Results

### Baseline characteristics

The mean age of the 21,308 Korean participants was 49.5 ± 16.1 years (42.9% male). The baseline characteristics of the study participants, according to ALP quartiles, are noted in Table 1. Higher serum ALP was associated with older age and the male sex (P<0.001), as well as higher BMI, waist circumference, BP, AST, ALT, glucose, cholesterol, triglyceride, and insulin. Higher ALP quartiles showed higher prevalence of increased eGFR (>130 ml/min/1.73m^2^) and reduced eGFR group (<60 ml/min/1.73m^2^) stratified by age (S1 Table). Higher ALP levels were also associated with lower HDL levels (P<0.001). In addition, ferritin level and WBC count were associated with higher ALP levels (P<0.001). There was a higher prevalence of DM, HTN, MI, angina, stroke, malignancy, smoking, and alcohol consumption, in the participants in the highest versus the lowest quartiles of serum ALP (P≤0.008) (Table 1).

**Table 1:** Baseline characteristics stratified by serum alkaline phosphatase (ALP) quartiles.

	ALP quartiles				
	<176 (N = 5274)	176–214 (N = 5340)	215–261 (N = 5372)	>262 (N = 5322)	*P*
ALP (IU/L)	148.2±20.6	195.0±11.3	236.7±13.4	318.8±62.7	<0.001
Age (years)	43.2±14.2	47.7±15.8	51.1±15.9	56.1±15.8	<0.001
Men (%)	1141 (26.8)	2405 (45.0)	2811 (52.3)	2513 (47.2)	<0.001
BMI (kg/m^2^)	22.9±3.2	23.6±3.3	23.9±3.4	24.0±3.4	<0.001
Waist circumference (cm)	77.7±9.8	81.0±9.8	82.6±9.7	83.3±9.7	<0.001
SBP (mmHg)	113.0±15.8	118.3±17.0	121.3±17.2	124.9±18.1	<0.001
DBP (mmHg)	73.7±10.2	76.4±10.6	77.8±10.7	78.3±11.0	<0.001
Hemoglobin (g/dL)	13.4±1.5	13.9±1.6	14.2±1.6	14.1±1.6	<0.001
WBC count (thous/uL)	5.7±1.5	6.0±1.6	6.2±1.7	6.3±1.8	<0.001
AST (IU/L)	19.2±7.9	21.2±8.8	23.0±10.4	25.6±18.8	<0.001
ALT (IU/L)	16.7±10.7	20.2±13.9	22.8±15.4	25.0±22.9	<0.001
Glucose (mg/dL)	93.3±15.6	96.2±18.6	98.4±22.7	103.1±30.6	<0.001
Cholesterol (mg/dL)	181.8±34.6	187.8±34.7	191.5±36.4	194.2 ±38.3	<0.001
TG (mg/dL)	108.3±81.8	129.9±93.5	145.9±113.1	156.5±140.0	<0.001
HDL (mg/dL)*	55.3±12.8	52.5±12.7	51.0±12.2	50.2±12.4	<0.001
Ferritin (ng/ml)	62.5±111.3	80.3±81.6	94.0±93.2	104.2±151.2	<0.001
25-OH vitamin D	17.9±6.5	18.5±6.7	19.0±7.0	18.8±6.9	<0.001
Proteinuria (%)†	28 (0.6)	55 (1.1)	64 (1.2)	111 (2.2)	<0.001
DM (%)	315 (6.4)	429 (8.6)	536 (10.5)	803 (15.9)	<0.001
HTN (%)	996 (19.7)	1532 (29.9)	1915 (36.5)	2281 (43.8)	<0.001
MI (%)	23 (0.4)	46 (0.9)	45 (0.8)	60 (1.1)	0.004
Angina (%)	76 (1.4)	93 (1.7)	96 (1.8)	128 (2.4)	0.008
Stroke (%)	59 (1.1)	96 (1.8)	109 (2.0)	158 (3.0)	<0.001
Malignancy (%)	99 (1.9)	85 (1.6)	154 (2.9)	214 (4.0)	<0.001
Smoking (%)					<0.001
Current	1179 (22.4)	1691 (31.7)	1927 (35.9)	1778 (33.5)	
Ex- or non-smoker	4090 (77.6)	3640 (68.3)	3443 (64.1)	3535 (66.5)	
Alcohol (%)	2410 (45.9)	2500 (47.1)	2421 (45.2)	1937 (36.5)	<0.001

Abbreviations: BMI (body mass index), systolic blood pressure (SBP), diastolic blood pressure (DBP), estimated glomerular filtration rate (eGFR), white blood cell (WBC), aspartate aminotransferase (AST), alanine aminotransferase (ALT), triglyceride (TG), high density lipoprotein (HDL), diabetes (DM), and hypertension (HTN).

*Levels of HDL cholesterol and insulin were measured in 15800 participants.

†Proteinuria was defined as dipstick urinalysis above 1+.

### Associations of serum alkaline phosphatase quartiles with metabolic syndrome according to estimated glomerular filtration rate

We divided participants into 4 groups, according to eGFR (>120, 90–119, 60–89, and <60 ml/min/1.73 m^2^). The associations with MS were analyzed according to ALP quartiles in each eGFR group, adjusted by multiple factors. The prevalence of MS was 14.1% in the analyzed participants. The highest ALP quartiles were noted in patients with the highest risk of MS in each eGFR group (P<0.001). However, ALP quartiles were not related to MS in participants with eGFR <60 ml/min/1.73 m^2^ (P = 0.422) Furthermore, the participants with eGFR >120 ml/min/1.73 m^2^ showed the highest risk (3.848, 95% CI 1.876–7.892) for MS, in the highest ALP quartiles, compared with the lowest quartile. The highest quartile of eGFR 90–119 ml/min/1.73 m^2^ showed a 1.899-fold risk, compared with the lowest quartile (95% CI, 1.504–2.399), and the eGFR 60–89 ml/min/1.73 m^2^ quartile showed a 1.637-fold risk (95% CI, 1.283–2.087) (Table 2).

**Table 2:** Associations between serum alkaline phosphatase quartiles and metabolic syndrome according to the estimated glomerular filtration rate (eGFR).

	ALP quartiles*	OR (95% CI)		
	<176 (IU/L)	176–214 (IU/L)	215–261 (IU/L)	>262 (IU/L)
eGFR(ml/min/1.73m^2^)				
>120	reference	1.979	3.357	3.848
		(0.931–4.204)	(1.615–6.979)	(1.876–7.892)
90–119	reference	1.244	1.583	1.899
		(0.974–1.589)	(1.254–2.000)	(1.504–2.399)
60–89	reference	1.327	1.525	1.637
		(1.029–1.712)	(1.192–1.951)	(1.283–2.087)
<60	reference	1.625	1.020	1.450
		(0.727–3.630)	(0.462–2.251)	(0.697–3.013)

*The odds ratios of serum alkaline phosphatase quartiles were adjusted by age, sex, hemoglobin, cholesterol, current smoking, alcohol use, hypertension, myocardial infarction, stroke, and malignancy.

### Associations of serum alkaline phosphatase quartiles with diabetes according to estimated glomerular filtration rate

The associations with DM were analyzed among ALP quartiles according to the eGFR group, adjusted by multiple factors. In participants with eGFR ≥60 ml/min/1.73 m^2^, the highest quartile showed a significantly increased risk for DM, compared with the lowest quartile (P<0.05). The participants with eGFR >120 ml/min/1.73 m^2^ showed the highest risk (2.166, 95% CI, 1.084–4.329) for DM, compared with the lowest quartile. The highest quartile of eGFR 90–119 ml/min/1.73 m^2^ showed a 1.355-fold risk, compared with the lowest quartile (95% CI, 1.073–1.711), and the eGFR 60–89 ml/min/1.73 m^2^ quartile showed a 1.260-fold risk (95% CI, 1.003–1.582) (Table 3).

**Table 3:** Associations between serum alkaline phosphatase quartiles and diabetes according to the estimated glomerular filtration rate (eGFR).

	ALP quartiles*	OR (95% CI)		
	<176 (IU/L)	176–214 (IU/L)	215–261 (IU/L)	>262 (IU/L)
eGFR (ml/min/1.73m^2^)				
>120	reference	1.271	1.238	2.166
		(0.610–2.646)	(0.581–2.640)	(1.084–4.329)
90–119	reference	0.809	0.913	1.355
		(0.626–1.047)	(0.715–1.164)	(1.073–1.711)
60–89	reference	1.099	1.106	1.260
		(0.866–1.396)	(0.875–1.397)	(1.003–1.582)
<60	reference	0.455	0.544	0.699
		(0.229–0.902)	(0.293–1.012)	(0.375–1.197)

*The odds ratios of serum alkaline phosphatase quartiles were adjusted by age, sex, systolic blood pressure, body mass index, hemoglobin, cholesterol, current smoking, alcohol, hypertension, myocardial infarction, stroke, and malignancy.

### Associations of albuminuria among serum alkaline phosphatase quartiles in participants with eGFR ≥ 60 ml/min/1.73m^2^

**Fig 1:**
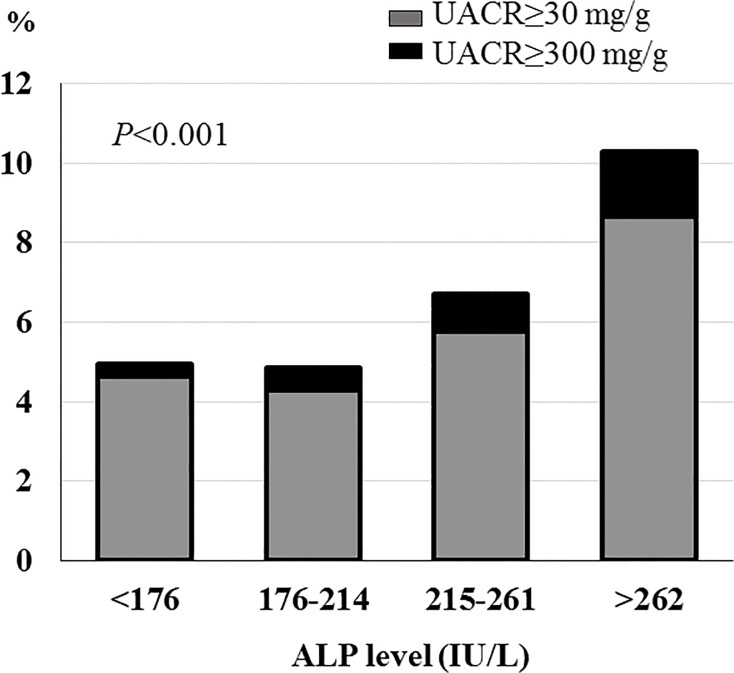
Associations of albuminuria among serum alkaline phosphatase quartiles in participants with eGFR ≥ 60 ml/min/1.73m^2^.

UACR was evaluated in 4883 participants with eGFR ≥ 60 ml/min/1.73m^2^. Higher ALP quartiles showed higher prevalence of albuminuria (*P*<0.001) (Fig 1). After adjustment of age and sex., the ALP quartiles were significantly associated with albuminuria in participants with eGFR ≥ 60 ml/min/1.73m^2^. The highest ALP quartile had a 1.631-fold risk elevation for albuminuria (95% CI, 1.158–2.297, P = 0.005).

### Associations of renal hyperfiltration among serum alkaline phosphatase quartiles in participants with eGFR ≥ 60 ml/min/1.73m^2^

**Fig 2:**
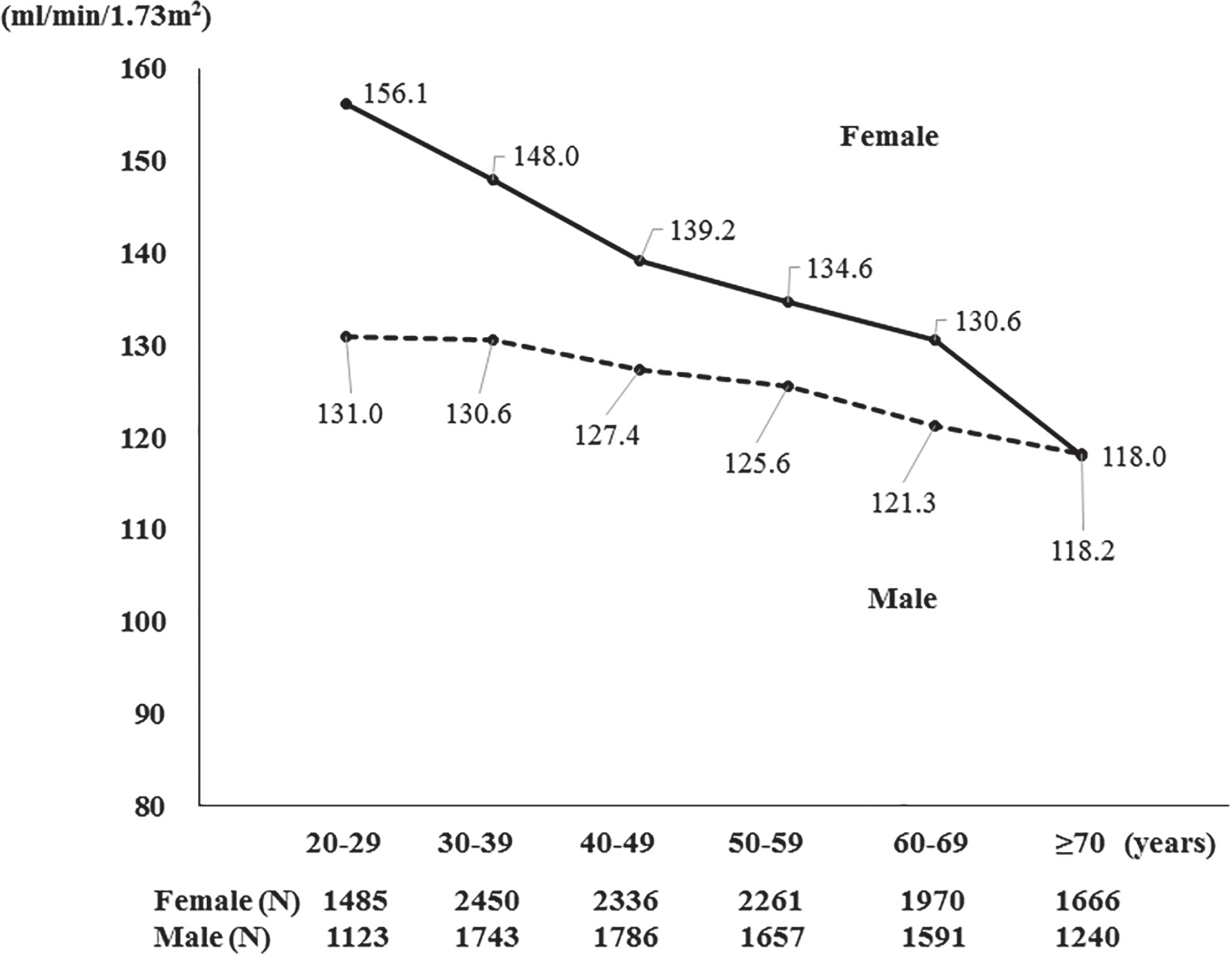
Distribution of estimated glomerular filtration rate by sex and age. The 97.5th percentiles are shown in 10-year age groups. Hyperfiltration was defined as an estimated glomerular filtration rate (eGFR) over the age- and sex-specific 97.5th percentile.

Renal hyperfiltration was defined as eGFR exceeding the age- and sex-specific 97.5^th^ percentile. The reference values for hyperfiltration for each 10-year age group are shown in Fig 2. We evaluate the associations between hyperfiltration and ALP quartiles, in participants with eGFR ≥ 60 ml/min/1.73 m^2^. After adjustment, the highest ALP quartile had a 1.624-fold risk elevation for hyperfiltration (95% CI, 1.204–2.192, P = 0.002). In addition, hyperfiltration was significantly associated with hemoglobin, triglyceride, WBC, DM, smoking, and alcohol consumption (P<0.05) (Table 4).

**Table 4:** Associations of glomerular hyperfiltration among serum alkaline phosphatase quartiles in participants with an estimated glomerular filtration rate ≥ 60 ml/min/1.73 m^2^.

	OR	95% CI	P
Serum ALP quartiles*			
<176 (IU/L)	reference		0.003
176–214 (IU/L)	1.254	0.939–1.675	0.126
215–261 (IU/L)	1.046	0.767–1.427	0.775
>262 (IU/L)	1.624	1.204–2.192	0.002
Hemoglobin	0.787	0.726–0.853	<0.001
Triglyceride	1.002	1.001–1.002	<0.001
WBC	1.109	1.048–1.173	<0.001
DM	1.516	1.017–2.261	0.041
Current smoking	1.466	1.124–1.912	0.005
Alcohol	1.347	1.076–1.685	0.009

*The odds ratios of serum alkaline phosphatase quartiles were adjusted by age, sex, diastolic blood pressure, glucose, body mass index, alanine aminotransferase, aspartate aminotransferase, hemoglobin, white blood cell count (WBC), ferritin, triglyceride, diabetes (DM), metabolic syndrome, angina, current smoking, and alcohol consumption.

## Discussion

Higher ALP level was associated with renal hyperfiltration in our nation-wide population based study. Serum ALP was significantly related to DM and MS in participants with eGFR > 60ml/min/1,73m^2^, and these relationships were stronger in participants with an upper-normal range of eGFR.

ALP is present in a variety of organisms, from bacteria to humans. Four isoenzymes of ALP have been identified thus far, demonstrating its varied functions: placental, germ cell, intestinal, and tissue-non-specific (liver/bone/kidney) ALP [17]. In the kidney, ALP is expressed on the brush border membranes of proximal tubular cells. The two isoenzymes, tissue-non-specific ALP and intestinal ALP, are expressed in the entire proximal tubule and the lower tubular segment, respectively [18,19]. In patients with reduced renal function, high serum ALP was associated with chronic kidney disease, mineral bone disorder, secondary hyperparathyroidism, heart failure, vascular calcification, and death [8,20,21,22,23]. However, little was known about ALP in individuals with an upper-normal range of renal function.

In sepsis-induced acute kidney injury, urine ALP levels are elevated, accompanied by loss of brush border membrane during endotoxemia [24]. The urinary ALP excretion is increased by the remnant kidney after contralateral nephrectomy in kidney donors, and might be involved in increased metabolism by renal tubular cells, in individuals with renal hyperfiltration [11]. In this study, a strong association was identified between metabolic disorders and serum ALP, alongside an increase of eGFR. In addition, the highest serum ALP quartile was an independent factor for predicting renal hyperfiltration.

Previous studies have evaluated the association between serum ALP levels and metabolic diseases such as MS and DM [9,10]. Tissue-non-specific ALP was considered to correlate with insulin resistance and MS [9,10,25]. Intestinal ALP is involved in lipid absorption across the enterocyte, and administration of intestinal ALP prevents MS, by the inhibition of endotoxin absorption [26]. Renal hypertrophy and hyperfiltration become manifest after the onset of type 1 diabetes, during the development of diabetic nephropathy [27]. Recent studies report that renal hyperfiltration is associated with proximal tubular epithelial hypertrophy and injury, accompanied by glomerular lesions [28,29]. Obesity leads to an increase in glomerular tuft area, mesangial expansion, renal lipid accumulation, and macrophage infiltration in the renal medulla [30]. Urine ALP is a marker of tubular damage and intravenous administration of ALP improved sepsis-related tubular injury [24,31]. In addition, urinary ALP was significantly higher in type 2 diabetics with nephropathy, and sensitive marker for diagnosis of diabetic nephropathy [32]. Although the mechanism underlying the link between serum ALP levels and hyperfiltration was unknown, it is hypothesized that elevated levels of serum ALP are associated with increased endogenous production, in response to tubular epithelial hypertrophy and injury.

Using the nationwide data obtained from the KNHANES IV-V (a large sample size within the general population) improved the strength of our study. Secondly, we used an identical method to measure serum ALP. Thirdly, to overcome the variances of eGFR seen in changes in age and sex, hyperfiltration was defined as eGFR above the age- and sex-specific 97.5^th^ percentile. Finally, we excluded participants with liver disease to avoid confounding factors.

Our study also had limitations. Firstly, despite being a commonly used marker of renal function, serum creatinine had a limitation to detect changes when renal function is normal or abnormally increased [14]. However, serum creatinine is the most commonly used marker detecting renal function in clinical setting, and we defined renal hyperfiltration by using strict criteria. Secondly, although we used an identical machine to measure serum creatinine, different reagents were used in KNHANES III and IV. However, subgroup analysis revealed that serum ALP levels were significantly higher in participants with hyperfiltration in both the KNHANES III and IV (data are not shown). Thirdly, isoforms of serum ALP were not analyzed. We could not investigate whether intestinal or liver/bone/kidney sources of ALP are associated with hyperfiltration. Finally, since our study was cross-sectional, causality cannot be proven. In conclusion, the results of this study suggest that the associations with serum ALP and metabolic disorders is stronger in participants with an upper-normal range of eGFR and that higher ALP levels are significantly associated with renal hyperfiltration in the general Korean population. Further studies are required to determine the mechanism involved in the association between elevated serum ALP levels and renal hyperfiltration.

## Supporting Information

S1 Table Estimated glomerular filtration rate stratified by age according to alkaline phosphatase quartiles. The highest ALP quartiles showed higher prevalence of increased eGFR (≥130 ml/min/1.73m^2^).
(DOCX)
