# Supplementary material for: Associations between Renal Hyperfiltration and Serum Alkaline Phosphatase
Source: PLoS One. 2015 Apr 8;10(4):e0122921. doi: 10.1371/journal.pone.0122921 (PMC4390244; doi:10.1371/journal.pone.0122921)
Supplement: S1 Table — The highest ALP quartiles showed higher prevalence of increased eGFR (≥130 ml/min/1.73m2). (DOCX) [file pone.0122921.s001.docx]

S1 table. Estimated glomerular filtration rate stratified by age according to alkaline phosphatase quartiles

|  | Serum alkaline phosphatase (ALP) (IU/L) quartiles | | | |
| --- | --- | --- | --- | --- |
|  | >262 (%) | 215-261 (%) | 176-214 (%) | <176 (%) |
| Age |  |  |  |  |
| 20-39 years* |  |  |  |  |
| eGFR≥130 | 89 (8.7) | 73 (4.9) | 129 (7.0) | 188 (7.7) |
| 60-129 | 931 (91.2) | 1420 (95.1) | 1723 (93.0) | 2243 (92.2) |
| <60 | 1 (0.1) | 0 | 1 (0.1) | 3 (0.1) |
| 40-59 years |  |  |  |  |
| eGFR≥130 | 55 (3.0) | 45 (2.2) | 56 (2.6) | 61 (3.0) |
| 60-129 | 1756 (96.0) | 2004 (97.1) | 2047 (96.8) | 1962 (96.5) |
| <60 | 19 (35.2) | 14 (25.9) | 11 (20.4) | 10 (18.5) |
| ≥6o years |  |  |  |  |
| eGFR≥130 | 52 (2.1) | 25 (1.4) | 22 (1.6) | 10 (1.2) |
| 60-129 | 2222 (89.9) | 1664 (91.6) | 1259 (91.7) | 726 (90.0) |
| <60 | 197 (8.0) | 127 (7.0) | 92 (6.7) | 71 (8.8) |

**P*<0.05
